# Effective Point Cloud Analysis Using Multi-Scale Features

**DOI:** 10.3390/s21165574

**Published:** 2021-08-19

**Authors:** Qiang Zheng, Jian Sun

**Affiliations:** 1 State Key Laboratory for Strength & Vibration, School of Aerospace, Xi’an Jiaotong University, Xi’an 710049, China; yongzhoucaomin@stu.xjtu.edu.cn; 2 Shaanxi Engineering Laboratory for Vibration Control of Aerospace Structures, Xi’an Jiaotong University, Xi’an 710049, China

**Keywords:** deep learning, point cloud, multi-scale, classification, part segmentation

## Abstract

Fully exploring the correlation of local features and their spatial distribution in point clouds is essential for feature modeling. This paper, inspired by convolutional neural networks (CNNs), explores the relationship between local patterns and point coordinates from a novel perspective and proposes a lightweight structure based on multi-scale features and a two-step fusion strategy. Specifically, local features of multi-scales and their spatial distribution can be regarded as independent features corresponding to different levels of geometric significance, which are extracted by multiple parallel branches and then merged on multiple levels. In this way, the proposed model generates a shape-level representation that contains rich local characteristics and the spatial relationship between them. Moreover, with the shared multi-layer perceptrons (MLPs) as basic operators, the proposed structure is so concise that it converges rapidly, and so we introduce the snapshot ensemble to improve performance further. The model is evaluated on classification and part segmentation tasks. The experiments prove that our model achieves on-par or better performance than previous state-of-the-art (SOTA) methods.

## 1. Introduction

As a typical data structure used in the three-dimensional (3D) domain, the point cloud has been widely used in actual scenes, such as for autonomous vehicles, robots, and computer graphics. Although it is convenient to use coordinates to represent point cloud data, this kind of disordered collection of points is an irregular data format. It does not have a regular structure similar to the image, making the further analysis of a point cloud a challenging task. Recently, as convolutional neural networks (CNNs) [1,2,3,4] have achieved great success on data in regular formats (such as images and videos), many works have tried to convert raw point cloud data into 3D voxels [5,6,7,8,9,10] or two-dimensional (2D) image collections [11,12,13,14,15] so that they can be further processed through CNN.

However, these methods have inherent shortcomings. For voxel-based methods, quantization errors may be introduced during voxelization, which may cause a potential loss of geometric information and degradation of shape details. Furthermore, the data are so sparse that their analysis is highly demanding in terms of storage and computing resources. Although accurate classification can be achieved for view-based methods, it is necessary to obtain multiple images from different perspectives to ensure that the generated 2D projection can contain sufficient geometric features of the surface of a 3D object, resulting in the loss of spatial information between the images and hindering semantic segmentation tasks. Therefore, it is necessary to develop a geometry learning method that directly operates on the point cloud and understands the relationship between local features and their spatial distribution.

PointNet [16] was the first deep learning-based method to manipulate point cloud data directly. It applies shared multi-layer perceptrons (MLPs) to learn the features of each point independently and then uses a symmetric function to aggregate the features of each point to output shape-level features. The limitation of PointNet [16] is that it directly aggregates all the point-wise features into one shape-level feature while ignoring the geometric features of local regions. PointNet++ [17], an advanced version of PointNet, is a hierarchical network structure with paralleled shared mini-PointNets to extract local features layer by layer in series of expanded perception fields. Following PointNet [16] and PointNet++ [17], the entire community is paying more attention to the exploration of the local features ignored by PointNet. DGCNN [18] summarizes previous works and proposes an inductive feature extraction module.

**Figure 1:**
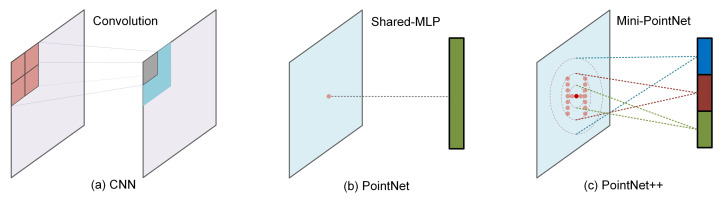
Three typical feature extraction layers. (**a**): Convolution layers in a convolutional neural network (CNN) implicitly contain the positions of local patterns. (**b**): PointNet only takes the coordinates as inputs and obtains the point-wise features that reflect the global distribution characteristics without local geometric information. (**c**): Mini-PointNet in PointNet++ only combines the local relative coordinates of neighborhood points and their features as inputs. PointNet++ adopts the multi-scale grouping (MSG) method to extract multi-scale features and directly concatenate the output features as the input of the following feature extraction module.

However, these point-based methods have intrinsic limitations. Because of the regular rigid structure, for classification tasks with CNNs in the 2D image domain, the local patterns extracted at specific positions have coherence in the position distribution in feature maps. This coherence shows that the regular grid structure implicitly contains the distribution of local features, and the features extracted by CNN are essentially a combination of local features and the positions implied by the grid index (see Figure 1a). PointNet [16] focuses on the point-wise features, and the local features are entirely ignored (see Figure 1b). In PointNet++ [17], the global coordinates are only used to sample and group the neighboring points, and only the local relative coordinates without global distribution information are concatenated with features for subsequent inference, limiting further improvements to performance (see Figure 1c). DGCNN [18] directly mixes the global coordinates and relative local features and does not make a substantial distinction between the roles of the two characteristics. Based on the above analysis, we reinterpret the relationship between local features and global coordinates. Local features are derived from the local relative coordinates, which reflect the geometric features of the local regions and should not be affected by the locations. In other words, local features and global coordinates are two independent features of a point cloud that should be extracted separately.

**Figure 2:**
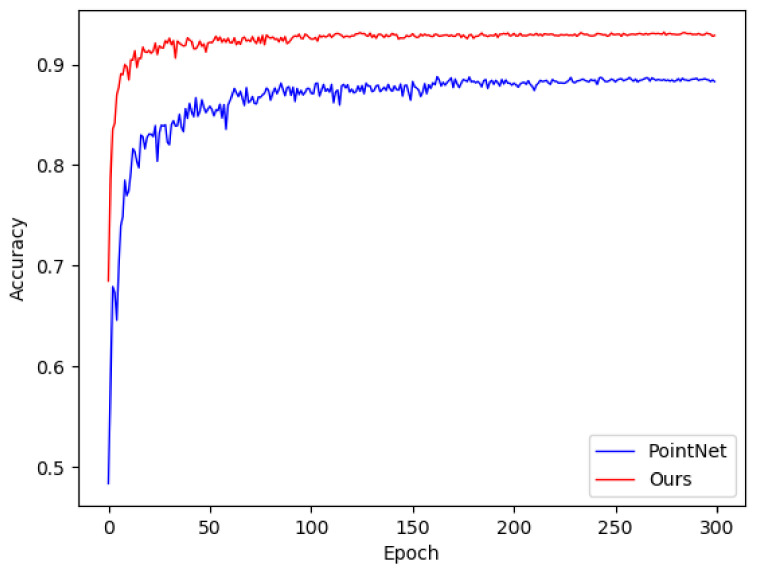
Accuracy curves with exponential decay learning schedule. The curve of PointNet is also illustrated for reference, and the proposed model maintains higher accuracy and converges rapidly.

Furthermore, the size of the neighborhood is a crucial parameter that directly affects the performance of deep networks. Different scales correspond to different geometric features. Combining these features to obtain more representative characteristics is a topic that is worthy of consideration. Previous models such as the classical PointNet++ [17] adopt multi-scale grouping (MSG) (see Figure 1c) and multi-resolution grouping (MRG) to improve performance. In each feature learning module, features of different scales are concatenated together, which means that in the forward inference, the features of various scales are always mixed together. These features of different geometric characteristics may exert adverse effects on each other, thus limiting the performance improvement. In the Inception layer [19], convolution kernels of different scales are applied to extract features corresponding to different receptive fields in parallel and then combined to improve performance. Inspired by Inception [19], we design a lightweight structure that applies independent branches to extract the local features of multiple scales, respectively, and fuse them with the shared global distribution feature to obtain multiple composite features. These composite features are further aggregated to obtain a representative shape-level feature. Moreover, our model shows rapid convergence during inference (see Figure 2), allowing us to introduce the snapshot ensemble [20] to improve performance without additional computational cost.

The main contributions of this paper are summarized as follows:

- We study the relationship between global coordinates and local features and understand the role of global coordinates in point cloud analysis from a novel perspective;
- We propose a lightweight structure based on multi-scale features and a two-step fusion strategy for effective feature learning;
- We introduce the snapshot ensemble to improve performance without additional computational cost;
- We conduct extensive experiments on challenging benchmarks across various tasks and achieve outstanding performance compared with previous state-of-the-art (SOTA) methods.

## 2. Related Work

The previous point cloud analysis methods can be mainly summarized into three categories: voxel-based methods, view-based methods, and point-based methods.

### 2.1. Voxel-Based Methods

Voxelization converts irregular 3D point cloud data into a standard 3D grid format so that 3D convolution operations based on 3D grid data can be conveniently used to process point cloud data. Thus, some advanced point cloud analyses can be realized by 3D CNN, such as classification and segmentation tasks. The performance of these methods has been evaluated in the computer vision community [5,6,7]. However, in the voxelization of the point cloud, an inevitable loss of shape information occurs. Besides, because the data size of voxels increases cubically with the resolution of the 3D voxel grid, it poses a massive challenge in terms of storage and computation resources. Therefore, in practical applications, the voxel data resolution is often constrained, exacerbating the loss of shape information. Several works have proposed that the computation on empty voxels can be skipped so that higher resolution grids can be handled [8,9,10] and limitations regarding the resolution can be overcome. However, the loss of geometric information in the process of voxelization is still inevitable. To avoid the loss of shape information, methods that can directly process the point cloud have apparent advantages.

### 2.2. View-Based Methods

View-based methods project a point cloud into collections of 2D views and feed them into the CNN. Since the 2D CNN has achieved promising results in the computer vision community, view-based methods have achieved great success in point cloud classification tasks [11,12,13,14]. However, the limitations of these methods should also be considered. When a complete 3D shape is represented as a set of discrete views, the spatial relationship between them is ignored, and it is not easy for this to be represented clearly. Therefore, view-based methods achieve dominant performance in classification tasks, but this is not trivial in tasks such as scene segmentation because part of the spatial information of the point cloud is lost during 2D projection. Besides, to obtain accurate classification, it is often necessary to capture these views from as many perspectives as possible and use them for training [15], which leads to a significant increase in the demand for storage and computation resources.

### 2.3. Point-Based Methods

In contrast to the methods mentioned above of transforming point cloud data into other formats, point-based methods have recently been developed to process 3D point clouds directly. PointNet [16] is the pioneering work in this category, which uses shared MLPs to independently extract point-wise features and then aggregates them into global features through a symmetric function (max pooling). PointNet++ [17] is an improved version of PointNet. PointNet++ [17] introduces a hierarchical structure to extract abstract local features and finally outputs shape-level features with gradually expanded receptive fields layer by layer. Since the point cloud data are not transformed, the quantization errors caused by the voxel-based methods and the massive storage and computation costs caused by the view-based methods are fundamentally eliminated. Of the point-based methods that have been developed [16,17,21,22,23,24,25], DGCNN [18] systematically summarizes the general form of EdgeConv and is regarded as a milestone work in the field. Their work clearly shows that many previous point-based methods, including PointNet [16] and PointNet++ [17], can be regarded as special cases of EdgeConv under certain assumptions. PointWeb [24] enriches point features by weighting all edges in a local area using point-pair differences, which improves the discrimination of features. PCCN [26] proposes parametric continuous convolution, which develops parameterized kernel functions defined on the point cloud, making the convolution kernel adapt to the point cloud’s geometric characteristics. PointConv [27] extends the dynamic filter to a convolution-like operation and builds a deep convolution network for point cloud analysis. The A-CNN [28] defines the orders of points in annular regions and applies annular convolutions to capture each point’s local geometric features. There are also some works that focus on local patterns and their distribution characteristics. The shape-oriented CNN [29] simultaneously learns the underlying local shapes and the long-ranged dependencies between them. Point2SpatialCapsule [30] learns not only geometric features but also the relationship between local regions. Point2Node [31] extracts features by integrating relations of nodes with self, local, and non-local nodes in high-dimensional graphs. SK-Net [32] jointly performs the inference of spatial keypoints and the learning of features, extracting the local patterns and the correlation between different regions. GS-Net [33] extracts features both in the Euclidean space and eigenvalue space, allowing it to aggregate both local and holistic features.

From the perspective of the emphasized feature species, PointNet [16] focuses on point-wise features, while PointNet++ [17], PointWeb [27], PointConv [27], PCCN [26], and the A-CNN [28] only focus on local features, limiting the representation of the extracted features. DGCNN [18], Shape-Oriented CNN [29], Point2SpatialCapsule [30], Point2Node [31], SK-Net [32], and GS-Net [33] simultaneously consider local features and spatial distribution characteristics and use different methods to extract these features. Specifically, DGCNN [18] dynamically builds graphs in Euclidean space and high-dimensional feature space; Point2Node [31] also involves graph operations in high-dimensional feature space. Shape-Oriented CNN [29], SK-Net [32], and GS-Net [33] use built-in modules containing complex operations to capture both local features and long-range dependence. Point2SpatialCapsule [30] includes the building of a large number of primary capsules and operations such as dynamic routing; these methods all pose a great demand on computing resources. From the perspective of whether multi-scale local features are considered, PointNet++ [17] and the A-CNN [28] extract multi-scale local features in each layer and directly concatenate them as the input of the next layer, directly mixing multi-scale features together without distinguishing the correlation and independence between them. Point2SpatialCapsule [30] directly transplants the multi-scale local feature extraction module of PointNet++ [17], so the same problem exists.

Compared with the above works, the method proposed in this paper has the following advantages:

- Only when the point cloud is input is a kNN search performed for each scale in the low-dimensional Euclidean space, avoiding the complicated graph operations in the high-dimensional feature space;
- This method uses the simplest shared MLP as the primary feature extraction method, making the network concise and efficient;
- This method simultaneously considers the intra-region relationship and inter-region relationship. Further, for the intra-region relationship, the multi-scale local features and especially their independence are fully considered. Thus, a more effective two-step fusion strategy is designed, including the fusion of each scale of local features, the global distribution characteristics, and the fusion of multi-scale features. The fusion process uses basic operations such as summation and averaging, avoiding complex embedded modules.

The main disadvantage of this method is that, because there is no down-sampling operation and the network extracts local features for all the input points, there are overlapping areas between adjacent centroids, resulting in the redundancy of computation.

## 3. Methodology

Our work can be regarded as an extension of PointNet [16] based on the same feature extraction method of shared MLPs. In this section, we first display the features of the different scales involved in the proposed structure and the two-step fusion strategy to integrate them effectively and then revisit the snapshot ensemble strategy briefly. Finally, we illustrate the network architecture in detail.

### 3.1. Feature Extraction

A point cloud is denoted as a set of unordered points, denoted as P={ p1,p2,…,pN}. Each point can be represented by a (*d* + *C*)-dim vector, in which *d* corresponds to a coordinate and *C* is associated with an additional feature, such as normal and RGB. In our work, we only take the coordinates of each point as inputs to validate the effectiveness of our structure for point cloud analysis.

#### 3.1.1. Multi-Scale Local Feature

Local features represent the local geometric characteristics around a certain point, which is not relevant to the position in which it is located. Thus, the extraction of local features should be independent of global distribution features. Moreover, the size of the local area is a critical argument for the performance of deep networks. For a small region in which the points are sparse and much randomness is introduced, learning the general patterns shared by broad masses of objects is challenging. In contrast, for a large region, the extracted local features may be restricted to an individual object, which leads to overfitting. Thus, we design a multi-scale local feature extraction method so that the network can automatically learn the local features and combine them effectively, thereby improving the generalization.

In the experimental part of this paper, the experimental results for a series of single-scale neighborhoods show that the recognition performances of these scales are not equal. This shows that the network extracts different features at various scales. For a specific object, a network may extract shape-sensitive features from some scales conducive to recognition while extracting degraded features from other scales that affect correct recognition. Therefore, simply mixing these features, such as directly concatenating them together on multiple layers like PointNet++ [17], does not necessarily bring about a significant performance improvement. The fusion of these features should first ensure the independence of these features in the process of extraction to retain the unique role of the features corresponding to each scale for recognition as much as possible.

Thus, we design multiple parallel branches to extract features based on local relative coordinates at each scale. A mini-PointNet composed of shared MLPs, which is used in PointNet++ for local feature extraction, is adopted in each branch. In addition, the mini-PointNets adopted in these branches are also shared, which effectively reduces the number of parameters and makes the structure concise.

#### 3.1.2. Global Distribution Feature

The coordinates provided by the raw point cloud represent the positions of these points, which act as the centroids of local regions. Hence, the features extracted from coordinates contain the spatial information of the regional patterns, which plays an essential role in effectively aggregating discrete multi-scale features. Compared with the local features based on relative coordinates, the distribution feature is essentially a feature with different receptive fields. Therefore, another independent branch that does not share the parameters of the multi-scale units is used to extract the global distribution characteristics corresponding to the coordinates. In the proposed model, shared MLPs are directly applied to extract the point-wise features, similar to the variant of PointNet without massive T-Nets.

#### 3.1.3. Fusion Strategy

Assuming that there are *M* local scales, we obtain a total of *M* local features and one shared distribution feature through *M* + 1 independent branches. The local features are generated from local coordinates and are irrelevant to the positions in which they are located. The point-wise features are generated from global coordinates, reflecting the spatial distribution of the regional patterns. The two kinds of features are independent and complementary to each other. Thus, we design a two-step fusion strategy for aggregating them effectively. In the first step, the shared global distribution feature and local features of *M* scales are added separately to obtain *M* mixed feature vectors; then, they are further fused through *M* stacked shared MLPs in parallel so that the *M* composite features are generated. Moreover, in the second step, the *M* mixed features undergo averaging and max-pooling operations to produce a shape-level feature with a great deal of shape awareness and robustness.

### 3.2. Snapshot Ensemble

The snapshot ensemble [20] is an efficient ensemble strategy that can achieve a batch of local suboptimal models in one training session without additional computation cost. Correspondingly, the cyclic cosine annealing learning rate is adopted so that the network can repeatedly converge to various local minima. The experiment proves that the proposed structure converges rapidly with the conventional exponential decay learning rate, which implies that the performance improvement is minimal after the initial rapid increase in accuracy, resulting in unnecessary computation cost (see Figure 2). The snapshot ensemble is introduced to address this limitation and take full advantage of the rapid convergence to improve performance further.

### 3.3. Network Architecture

**Figure 3:**
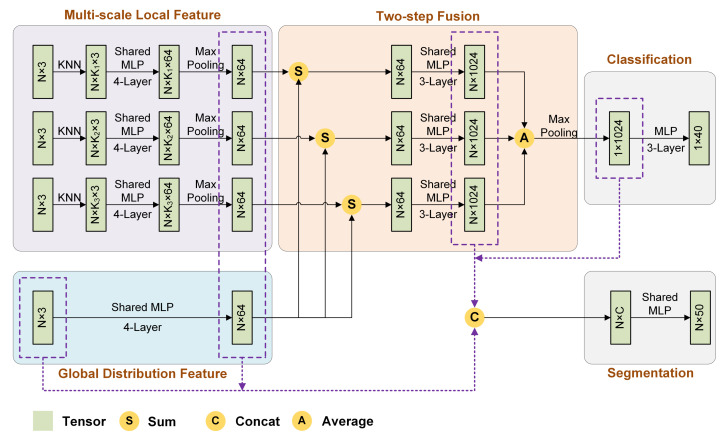
Network architecture.

As shown in Figure 3, the proposed architecture mainly contains four modules. The “multi-scale local feature” module applies multiple weight-shared branches to extract multi-scale features in parallel. The “global distribution feature” module is used to extract the global distribution characteristics shared by each local scale. The local features of each scale are added with the global feature and then fed into the “two-step fusion” module to be further transformed. Like the “multi-scale local feature” module, the fusion module comprises several parallel branches with shared weights and generates multiple abstract features. Then, these features are averaged, and a max-pooling operation is performed to fuse them further; finally, a shape-level feature with high representativeness is obtained. The remaining “classification” or “segmentation” module is used for classification or segmentation tasks and outputs predictions.

For the feature extraction, the entire network first extracts the features of different scales independently, including local regions and global distribution, and then performs a two-step fusion strategy. For the basic operators contained in the forward inference of the model, except the classification module, the network mainly performs shared MLPs, which are simple but effective. For spatial complexity, we adopt massive shared MLPs and extend the range of weight sharing to multiple spans between points, local regions, and branches, which can significantly reduce the number of parameters. All these characteristics make the structure lightweight and efficient.

### 3.4. Implementation Details

Our model was implemented with TensorFlow on an NVIDIA TITAN Xp GPU. For the cosine annealing learning rate, the initial value was 0.001, and the clip value was 0.000001, respectively, with a cycle period of 26 epochs. For the momentum of BN, the value began at 0.5 and decayed with a rate of 0.8. We used the Adam optimizer, and the batch size was 32. In addition, the multi-scale local region was set to (16, 32, 48, 64) for 2D Mixed National Institute of Standards and Technology database (MNIST) classification and (12, 24, 36, 48) for both ModelNet40 classification and segmentation tasks.

## 4. Experimental Results and Discussion

In this section, we describe the implementation of our approach for classification and segmentation tasks on challenging benchmarks to evaluate the performance of the proposed structure. Then, we describe the ablation studies conducted to research the design of the architecture. Finally, we evaluate the model’s complexity.

### 4.1. Point Cloud Analysis

#### 4.1.1. 2D Mnist Classification

**Figure 4:**
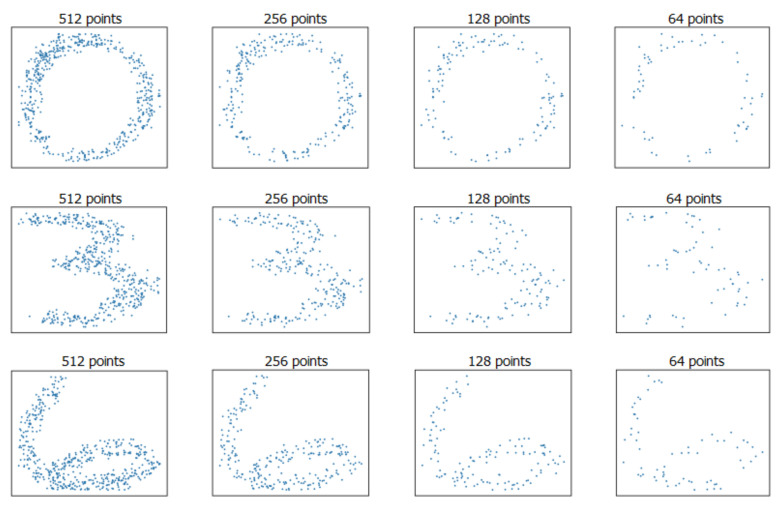
Examples of 2D MNIST point clouds at different densities.

The performance of the proposed model was first evaluated on 2D MNIST classification tasks. MNIST is a data set containing handwritten digital pictures, of which 60,000 are used for training and 10,000 are used for testing. For this experiment, each image was first converted into a 2D point cloud. Unlike previous methods, in the conversion process, we regarded gray values of the images as a probability distribution on the 2D area and sampled a fixed number of points on the image strictly according to the probability values, Figure 4 illustrates several examples of 2D MNIST point clouds with different densities. In addition, identical to the distribution of the 3D point cloud on a closed 2D surface, the points that were effective for 2D classification were mainly distributed on the 1D envelope curve; that is, points on the outline of handwritten digits. If a group of neighborhood points was entirely inside the contour, the extracted local features would have little effect on the recognition. Therefore, in this experiment, we increased the neighborhood size to ensure that the contour points could be included as much as possible. The results are shown in Table 1, and the proposed method achieved comparable performance with the 2D MNIST classification methods. Table 1 also displays the classification accuracy at different densities, proving that the proposed method can still extract features effectively even with extremely sparse points.

#### 4.1.2. 3D Modelnet40 Classification

The performance on shape classification tasks was evaluated on the ModelNet40 benchmark, which contains 13,834 CAD models from 40 categories and is split into 9843 models for training and 2468 models for testing. In total, 1024 points were uniformly sampled on mesh faces of each shape and then normalized into a unit sphere. Since sensors cannot directly obtain the normals on an object in actual scenarios, to evaluate the model’s performance for point cloud analysis and compare it fairly with other methods, we only used the coordinates as inputs.

The results are shown in Table 2. Our method is compared with previous point-based methods in the classification tasks on the ModelNet40 dataset. With the simple shared MLPs, the proposed structure outperforms all the other methods with only 1024 points as input. Note that RS-CNN [23] can achieve a higher accuracy of 93.6% with regard to 92.9% using a difficult 10-voting strategy (the best of 300 repeated tests), so we take 92.9% without voting as the benchmark result of RS-CNN [23] for a fair comparison. We also illustrate the results of sparser points. When the numbers of points were 256 and 512, the accuracies were 92.9% and 92.0%, respectively, outperforming many methods that take 1024 or more points as input. The results show that even if the simple shared MLPs are used as the primary feature extractors, our model obtains shape-awareness features by designing an effective feature extraction and fusion strategy.

#### 4.1.3. Part Segmentation

**Figure 5:**
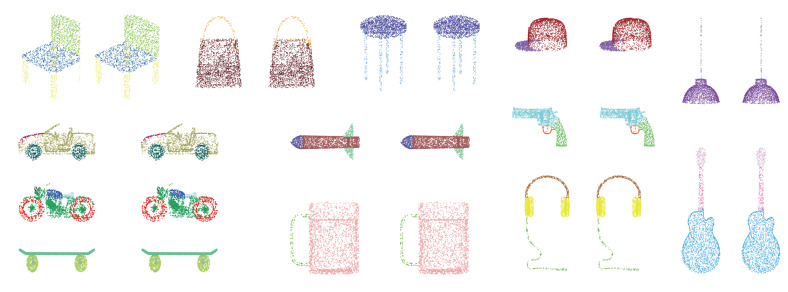
Segmentation examples on ShapeNet. For each example, left: ground truth, right: prediction.

We evaluated the use of our method for part-segmentation tasks on the ShapeNet-part dataset, which contains 16,881 shapes from 16 categories with 50 labeled parts in total. For each shape in the dataset, 2048 points were sampled, and most shapes were composed of less than six parts. In line with previous work with Pointnet [16], we split this dataset into two parts: one for training with 14,034 shapes and the other for testing with 2847 shapes. The mean Intersection over Union (IoU) metric was used to evaluate the segmentation performance on each point. The overall instance IoU (“Instance”) was computed by averaging IoUs over all the instances of test data. The class-wise IoU was the average of all IoUs corresponding to each category, and the mean category IoU (“Class”) was the mean of all the class-wise IoUs. The segmentation results are presented in Table 2. For a part segmentation with 2048 points, the local scales should have been set to (24, 48, 72, 96)—twice as much as the scales of (12, 24, 36, 48) with 1024 points in ModelNet40 classification tasks. Nevertheless, considering the computational efficiency, we still set the multi-region to (12, 24, 36, 48), which may have had a potential negative impact on the performance. Even so, our model still achieved comparable performance with the other methods. Figure 5 illustrates some qualitative segmentation results on the ShapeNet part benchmark.

### 4.2. Ablation Studies

#### 4.2.1. Architecture Analysis

The key to the success of our structure was the multi-scale feature extraction and the multi-level fusion strategy. Ablation experiments were conducted to evaluate the effectiveness of these components. All the experiments were conducted on the ModelNet40 dataset.

The results are shown in Table 3. All the experiments without “Global” show that the global distribution module was removed in the proposed structure. Similarly, lacking “Local” implies that the multi-scale features were not taken into account, and lacking “Fusion” means that the input 64-dim vector of the fusion module was directly averaged and sent to the max-pooling layer to get a shape-level feature. Furthermore, lacking “Cosine” indicates that the snapshot ensemble with cosine annealing strategy was discarded, and a conventional exponential decay learning schedule starting at 0.01 with a decay rate of 0.5 every 26 epochs (corresponding to one annealing cycle)was adopted.

For the model that only took multi-scale local features into consideration for classification (Model A), these features were simply a collection of disordered feature vectors without distribution characteristics, which could not be effectively fused to obtain shape-awareness features. In contrast, Model B only extracted the point-wise features without local geometric properties, restricting performance improvement. Furthermore, when Model A is compared with B, it can be concluded that local features were more discriminative than global distribution features, which is expected because the former contained many fine-grained features. Performance was severely degraded for Model C without the fusion strategy, proving that a reasonable feature fusion strategy to aggregate features from multiple scales is critical. Based on Model C, the two-step fusion strategy was introduced for Model D, and the performance was significantly improved from 86.8% to 93.0%, indicating the importance of the fusion strategy. Based on Model D, our model adopted the snapshot ensemble to improve the performance further and achieved a SOTA accuracy of 93.4%.

#### 4.2.2. Point Density

**Figure 6:**
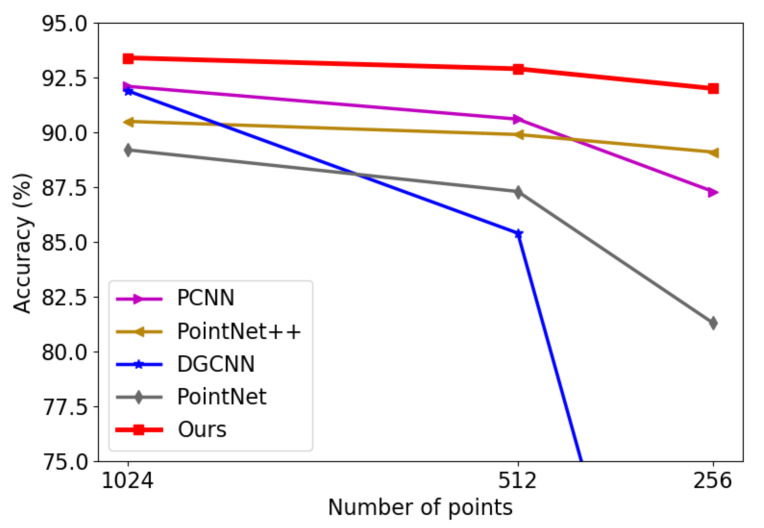
Results on sparser points.

The experiments above prove that the proposed structure can achieve outstanding performance with 1024 points, but the points are not always sufficiently dense in actual scenarios. Thus, the capacity to extract features with sparse points is vital. To evaluate the performance with sparse points, we randomly sampled points from the 2048 points. We set the numbers of points to 1024, 512, and 256, and set the multi-scale to (12, 24, 36, 48), (6, 12, 18, 24), and (3, 6, 9, 12) according to the proportional relationship to keep the local region sizes unchanged. The results are shown in Figure 6. Our model outperformed other methods at each density by a large margin. The result proves that our model can still extract discriminative features with the proposed feature fusion strategy, even with sparser points.

#### 4.2.3. Local Scale

The neighborhood size is a critical factor that influences the quality of learned features. To evaluate the impact of the local scale on performance, for 1024 points, we specified two groups of local scales: one group contained single-scales corresponding to 12, 24, 36, and 48, and the other group contained multi-scales corresponding to [12], [12, 24], [12, 24, 36], and [12, 24, 36, 48]. For 512 and 256 points, the local scale was set in the same way, but the size was reduced proportionally. The results are shown in Table 4. It can be seen that, for single-scale cases, the performance improved with the expansion of the scale in a specific scale interval. For multi-scale cases, as the scale and the number of scales increased, the accuracy also increased steadily. In addition, the comparison between a single scale and multiple scales of the same size shows that when the scale was small, such as 24 and [12, 24], 12 and [6, 12], the multi-scale caused a performance degradation, which implies that multi-scale does not always present advantages over single-scale. On the contrary, when the scale is large, the performance improvement of multiple scales exceeded that of a single scale. This effect was evident in the case of 1024 points and weaker in 512 and 256 points. However, even when the density or scale changed, the model could still maintain high accuracy, proving that the proposed structure is robust to density and scale changes. In particular, if the results corresponding to 256 points are compared with those of models B and C with 1024 points in the ablation experiments, it is evident that our model could maintain better performance by performing multi-scale feature extraction and a two-step fusion strategy.

#### 4.2.4. Reduced Training Dataset

Sufficient labeled samples are not always available, so it is critical to learn discriminative features with reduced training data. The point cloud version of the ModelNet40 training data provided by PointNet [16] consists of 5 files: the first four files each contain 2048 samples, and the last one contains 1648 samples. We directly treated each file as reduced training data, and the results are shown in Table 5. We also evaluated the performance of PointNet [16] for reference. Our model still worked and maintained a performance target even exceeded that of PointNet [16] trained with complete training data.

### 4.3. Complexity Analysis

Table 6 illustrates the space (number of parameters) and time (floating-point operations per sample) complexity of our model for the ModelNet40 classification with 1024 points. Due to the adoption of shared MLPs as the primary operation, our model was the most concise approach, with only about 1 million parameters. Notably, compared with PointNet [16] and PointNet++ [17], which also use shared MLPs for feature extraction, the number of parameters was reduced by 71.4% and 33.3%.

## 5. Conclusions and Future Work

This paper proposes a novel structure based on multi-scale feature extraction and a multi-level fusion strategy. Inspired by the CNN, we revisit the relationship between global coordinates and local patterns from a new perspective. This structure extracts multi-scale local features and global distribution characteristics, then fuses them with a two-step fusion strategy. The proposed model provides a practical framework using the simple shared MLP as the primary feature extraction method and achieves excellent performance. This framework is universally applicable, and more sophisticated feature extraction methods can be adopted instead of the shared MLP to improve the performance further. Moreover, our model shows rapid convergence and can be adapted to introduce the snapshot ensemble to improve performance significantly. Although effective, the use of shared MLPs to extract local features leads to the insufficient consideration of the fine-grained characteristics in local regions. In addition, extracting local features for all points results in overlapping points in adjacent local areas, which causes redundant information and extra computational consumption. More effective and efficient feature extractors will be further studied in the future.

## Figures and Tables

**Table 1:** Classification results on 2D MNIST classification (%) (“-”: unknown).

Method	Input	Accuracy
PointNet [16]	256	99.22
PointNet++ [17]	512	99.49
PointCNN [34]	160	99.54
Kd-Net [8]	-	99.10
Ours	64	96.79
Ours	128	98.82
Ours	256	99.29
Ours	512	99.52

**Table 2:** Shape classification and part segmentation results (%) (Mul: multi-scale, nor: normal, “-”: unknown, k: 1024).

	ModelNet40 (Classification)			ShapeNet (Segmentation)		
**Method**	**Input**	**Accuracy**	**Mul**	**Input**	**Cls. mIoU**	**Ins. IoU**
Pointwise-CNN [25]	1k	86.1		-	-	-
PointNet [16]	1k	89.2		2k	80.4	83.7
SCN [35]	1k	90.0		1k	81.8	84.6
Kd-Net(depth = 10) [8]	1k	90.6		4k	77.4	82.3
PointNet++ [17]	1k	90.7	✓	2k, nor	81.9	85.1
KCNet [36]	1k	91.0		2k	82.2	84.7
MSP-Net [37]	1k	91.7	✓	-	-	-
PointCNN [34]	1k	91.7		-	-	-
DGCNN [18]	1k	92.2		2k	82.3	85.1
PCNN [22]	1k	92.3		2k	81.8	85.1
MRFGAT [38]	1k	92.5	✓	-	-	-
Point2Sequence [39]	1k	92.6		-	-	-
A-CNN [28]	1k	92.6	✓	-	-	-
PointASNL [40]	1k	92.9		-	-	-
RS-CNN [23] w/o vot.	1k	92.9		2k	84.0	86.2
Point2Node [31]	1k	93.0		-	-	-
Point2SpatialCapsule [30]	1k	93.4	✓	2k	83.0	85.3
Ours	256	92.0	✓	-	-	-
Ours	512	92.9	✓	-	-	-
Ours (single scale{: 48)	1k	93.0		-	-	-
Ours	1k	93.4	✓	2k	81.8	85.1

**Table 3:** Ablation studies of our model (%).

Model	Local	Global	Fusion	Cosine	Accuracy
A	✓		✓		90.7
B		✓	✓		87.0
C	✓	✓			86.8
D	✓	✓	✓		93.0
Ours	✓	✓	✓	✓	93.4

**Table 4:** Results for different local scales (%).

	Single-Scale				Multi-Scale			
scale	12	24	36	48	(12)	(12, 24)	(12, 24, 36)	(12, 24, 36, 48)
Accuracy(1024)	92.4	92.8	93.1	93.0	92.4	92.6	93.1	93.4
scale	6	12	18	24	(6)	(6, 12)	(6, 12, 18)	(6, 12, 18, 24)
Accuracy(512)	91.4	92.4	92.3	92.9	91.4	92.1	92.5	92.9
scale	3	6	9	12	(3)	(3, 6)	(3, 6, 9)	(3, 6, 9, 12)
Accuracy(256)	88.2	90.8	91.7	91.9	88.2	90.7	91.5	92.2

**Table 5:** Results on reduced training set (%).

Method	File-0	File-1	File-2	File-3	File-4	All Files
PointNet [16]	79.2	81.8	81.2	79.3	78.8	89.2
Ours	89.5	90.0	89.0	89.5	88.3	93.4

**Table 6:** Complexity of our model in point cloud classification (M: million).

Method	#Params	#FLOPs/Sample
PointNet [16]	3.5 M	440 M
PointNet++ [17]	1.5 M	1684 M
PCNN [22]	8.2 M	294 M
Subvolume [12]	16.6 M	3633 M
MVCNN [11]	60.0 M	62,057 M
Ours	1.0 M	4372 M

## Data Availability

MNIST: http://yann.lecun.com/exdb/mnist/; ModelNet40: https://shapenet.cs.stanford.edu/media/modelnet40_ply_hdf5_2048.zip; ShapeNet part: https://cs.stanford.edu/~ericyi/project_page/part_annotation/index.html, all accessed on 19 July 2021.
